# Three Strains of *Tobacco etch virus* Distinctly Alter the Transcriptome of Apical Stem Tissue in *Capsicum annuum* during Infection

**DOI:** 10.3390/v13050741

**Published:** 2021-04-23

**Authors:** John F. Murphy, H. Tucker Hallmark, Thiruvarangan Ramaraj, Anitha Sundararajan, Faye Schilkey, Aaron M. Rashotte

**Affiliations:** 1 Department of Entomology & Plant Pathology, Auburn University, Auburn, AL 36849, USA; 2 Department of Biological Sciences, Auburn University, Auburn, AL 36849, USA; hth0003@tigermail.auburn.edu (H.T.H.); amr0008@auburn.edu (A.M.R.); 3 National Center for Genome Resources, Santa Fe, NM 87505, USA; tr@ncgr.org (T.R.); as@ncgr.org (A.S.); fds@ncgr.org (F.S.); 4 School of Computing, College of Computing & Digital Media, DePaul University, Chicago, IL 60604, USA

**Keywords:** *Tobacco etch virus*, *Capsicum annuum*, pepper, RNA-sequencing, transcriptome

## Abstract

*Tobacco etch virus* (TEV; genus *Potyvirus*) is flexuous rod shaped with a single molecule of single-stranded RNA and causes serious yield losses in species in the *Solanaceae*. Three TEV strains (HAT, Mex21, and N) are genetically distinct and cause different disease symptoms in plants. Here, a transcriptomic RNA sequencing approach was taken for each TEV strain to evaluate gene expression of the apical stem segment of pepper plants during two stages of disease development. Distinct profiles of Differentially Expressed Genes (DEGs) were identified for each TEV strain. DEG numbers increased with degree of symptom severity: 24 from HAT, 1190 from Mex21, and 4010 from N. At 7 days post-inoculation (dpi), when systemic symptoms were similar, there were few DEGs for HAT- and Mex21-infected plants, whereas N-infected plants had 2516 DEGs. DEG patterns from 7 to 14 dpi corresponded to severity of disease symptoms: milder disease with smaller DEG changes for HAT and Mex21 and severe disease with larger DEG changes for N. Strikingly, in each of these comparisons, there are very few overlapping DEGs among the TEV strains, including no overlapping DEGs between all three strains at 7 or 14 dpi.

## 1. Introduction

*Tobacco etch virus* (TEV) is a member of the genus *Potyvirus*, in the family *Potyviridae*, a genus among those with a large number of virus species [1]. TEV was first described by Johnson in 1930 (cited by [2]) and has since been shown to occur in North, Central and South America, Asia and Europe [3]. The host range for TEV includes 149 plant species in 19 families [4,5], although most hosts are in the *Solanaceae*.

TEV is a flexuous rod-shaped particle with a single molecule of single-stranded RNA genome that encodes 11 individual proteins translated as a polyprotein [6,7]. The 5′-terminus of the viral RNA has a VPg covalently linked by way of a tyrosine residue [8,9] and a 3′-terminus poly (A) tract [10]. The TEV RNA (HAT strain) sequence was reported by Allison et al. [11] and shown to consist of 9496 nucleotides able to translate a product of 3054 amino acids. The full-length RNA sequence for TEV strains HAT, Mex21 and N revealed 98% nucleotide sequence identity among strains Mex21 and N and 91% nucleotide sequence identity among all three TEV strains [12].

The three TEV strains differ in their pathogenicity in *Nicotiana* spp. and *Capsicum annuum* (bell pepper). In each of the different plant species, HAT induced mild disease symptoms, Mex21 moderate disease symptoms and N severe disease symptoms [12,13]. In bell pepper plants, symptom severity correlated with impacts on plant height and weight; however, accumulation of virus in systemically infected leaves was greater for HAT- than N-infected plants at 10 dpi and similar at 20, 30 and 40 dpi, despite the differences in impact on plant growth and development. Mex21 induced moderate disease severity; however, its accumulation in systemically infected leaves was significantly less than that of HAT and N throughout the experiments [13].

There are surprisingly few examinations of *C. annuum* at a transcriptome level even after the completion of the genome sequence in 2014 [14]. Of the transcriptome studies that have been completed, most focused primarily on fruit development or characteristics, capsaicin production or chilling stress [14,15,16,17]. In fact, there have been relatively few transcriptomic studies focused on virus infected pepper or other plants [18]. In pepper, large scale gene analysis examinations of viral infected plants have been limited to *Pepper golden mosaic virus* (PGMV), and *Cucumber mosaic virus* (CMV). While transcriptome examinations of TEV have been conducted, these have largely been limited to *Arabidopsis thaliana* [19,20]. These investigations have produced massive gene lists of viral-regulated genes, which encompass categories of stress, oxidative, and defense responses, chloroplast/photosynthesis processes, hormones, in addition to plant pathogen-related genes involved in PTI and ETI responses such as PRRs, MAPKs, and LRRs (for review see [18]).

Here, we present the first transcriptome data for three TEV strains infecting pepper plants. These TEV strains shown here and previously can induce differing degrees of disease severity in bell pepper plants. A transcriptomic RNA sequencing approach was taken to understand changes in gene expression of the apical stem segment of pepper plants at two stages in disease development. Many disease-related genes can be identified from these analyses. The numbers of differentially expressed genes in response to infection appear to correlate with TEV strain-specific disease severity in pepper plants.

## 2. Materials and Methods

### 2.1. Viruses, Plant Growth Conditions and Experimental Designs

TEV strains TEV-HAT, TEV-Mex21 and TEV-N were used in this study; they will be referred to as HAT, Mex21 and N, respectively. HAT was obtained from Dr. T. Pirone, University of Kentucky; Mex21 from Dr. M. Jahn, Cornell University (originally provided by Dr. L. Black, Louisiana State University) and N provided by Dr. B. Reddick, University of Tennessee. The HAT strain used in this study was the same strain referred to as HAT-AU by Velasquez et al. [12]. The TEV strains were maintained individually in *Nicotiana tabacum* L. ‘Kentucky 14’ by mechanical passage in a greenhouse (mean temperatures of 24 °C day and 20 °C night) at the Plant Science Research Center on the campus of Auburn University, AL, USA.

*Capsicum annuum* L cv. Calwonder (American Meadows, Williston, VT, USA) was used as the primary host for this study. Seed was sown in Sunshine Mix #8, soilless potting medium (Sun Gro Horticulture, Canada Ltd., Sacramento, CA, USA) in 72-well Styrofoam trays (Speedling Inc., Bushnell, FL, USA). Upon germination, seedlings were transplanted to 3.8-L round pots (one seedling per pot) containing Sunshine Mix #8. The growth medium was supplemented with slow-release fertilizer (18-6-12, Osmocote Classic, Scotts Company LLC, Marysville, OH, USA).

The experimental design consisted of rows made up of five plants, with each row consisting of a single virus treatment or buffer-mock inoculation control. The five-plant rows of treatments were randomized along a bench in the greenhouse. For inoculation of each virus treatment, the TEV strain was applied to leaves 1 and 2 (the two oldest true leaves along the main stem) by rub-inoculation when plants were at the 7 to 8-leaf stage of growth [21]. Virus inoculum consisted of systemically infected ‘Kentucky 14’ leaf tissue ground in 50 mM potassium phosphate buffer, pH 7.5. The buffer-mock inoculation treatment was performed in a similar manner using buffer without addition of tissue. The mortars and pestles as well as the buffer used for inoculation were chilled at 4 °C prior to use and kept on ice during the inoculation process.

### 2.2. Virus Infection Evaluations

Plant height measurements (in cm; *n* = 24, 22 and 18 per treatment at 0, 7 and 14 days post-inoculation, dpi) were taken from the soil-line to the apical tip of the main stem. The 0 dpi measurement was taken the day of inoculation.

Total plant RNAs were extracted from two Calwonder-infected plants from each TEV strain treatment and two buffer-mock control treatment plants at 7 and 14 dpi. At each time point, the two plants from each virus treatment were tested for virus accumulation in the stem by immuno-tissue blot analysis [21,22]. Tissue prints were generated by pressing the cut surface of the stem directly onto nitrocellulose membranes (Schleicher & Schuell, Keene, NH, USA). Each stem segment used to generate a tissue print was from a central position of the selected internode and included the internode immediately below inoculated leaves 1 and 2, the internode immediately above the inoculated leaves and an upper internode close to the apical portion of the stem. Tissue prints were allowed to dry at room temperature and stored at 4 °C until processed for virus detection as described previously [22].

### 2.3. Total RNA Isolation and High-Throughput Sequencing

For total RNA extractions, the upper most portion of the main stem from each plant subjected to immuno-tissue blot analysis, including the apical apex (ca. 0.5 cm in total), was excised and placed in a 1.5 mL Eppendorf tube. Total RNAs were extracted using a RNeasy Plant Kit (Qiagen, Valencia, CA, USA) according to manufacturer’s instructions. The apical stem segment was ground in 450 µL of RLT extraction solution (provided in the RNeasy kit) using a hand-held Teflon homogenizer. The final elution of RNAs from the purification column was carried out using 25 µL of water (provided with the RNeasy kit) and stored at −80 °C.

Total RNA was used for messenger RNA isolation with polyA selection and subsequent library construction with the TruSeq RNA sample preparation protocol from Illumina (San Diego, CA, USA). Two biological replicates were sequenced and analyzed for each of the TEV treatments and the buffer-mock control treatment as well as the two developmental time points (7 and 14 dpi). Single-end sequencing was performed on the 16 samples by the Illumina GAIIX platform, generating 333,589,860 1 × 54 bp reads. Raw sequence data are available for download at NCBI Sequence Read Archive under the BioProject ID: PRJNA476480, SRA accession: SRP150696.

### 2.4. Illumina mRNA Sequence Data Analysis

High quality sequence data generated for each of the 16 samples were aligned to the *C. annuum* cv CM334 genome downloaded from the Solgenomics webpage (ftp://ftp.solgenomics.net/genomes/Capsicum_annuum/, accessed on 1 November 2015) [23]. Additional alignment was made to GenBank accessions downloaded from NCBI for the TEV strains HAT-AU, Mex21, and N (GenBank accession number KM282187, KM282188, and KM282189, respectively, [12]. The associated annotation file, GFF format, was used to obtain genic information for downstream analysis. BAM alignments were generated using GSNAP (Genomic Short-read Nucleotide Alignment Program) (version released 2013_05_09) [24] with the following parameters; indel penalty = 2, maximum mismatches = 0.06, terminal threshold = 1000, novel splicing = 1, local splice distance = 10,000, distant splice penalty = 1000 and everything else set to default. Read counts were generated using NCGR’s in house pipeline, ALPHEUS [25]. Gene expression for each of the 16 samples was computed as a measure of the total number of reads uniquely aligning to the reference genome, binned by genic coordinates (information acquired from the annotation GFF3 file). Differential gene expression analysis was performed using the R [26] (http://www.R-project.org/, accessed on 1 November 2015) Bioconductor package DESEQ [27]. Raw read counts obtained were normalized to account for differences in sequencing depth and composition using methods implemented within the DESEQ package. Differential expression of pairwise comparisons (combinations of the different conditions) was assessed using the negative binomial test with the Benjamani–Hochberg false discovery rate (FDR) adjustment applied for multiple testing corrections. For this study, a FDR of 0.05 was applied and any candidate that had a *p*-adjusted value of ≤0.05 was considered to be significantly regulated. Lists of Differentially Express Genes (DEGs) for all TEV treatments vs. buffer-mock control at 7 and 14 dpi and comparisons of 7 vs. 14 dpi for individual treatments are given in Supplemental Tables S1 and S2. Examination of DEGs for connections to disease response as disease-related was conducted by searching gene descriptions using the key words disease, resistance, resistant, virus, and viral, while excluding DEGs from this list involved in drug-resistance as seen in Supplemental Table S3.

### 2.5. Confirmation of RNA-Sequencing by qPCR

Five genes identified as DEGs under stress conditions vs. buffer-mock control were selected to validate the RNA sequencing results using quantitative real-time PCR analysis (qPCR) following a modified protocol from Shi et al. [28]. Total RNA for each treatment was extracted as described above then used to generate cDNA for qPCR by reverse transcription using Quanta qScript cDNA supermix. qPCR was performed using PerfeCTa SYBR Green Supermix (QuantaBio, Beverly, MA) in 20 μL reactions on an Eppendorf realplex2 (Hamburg, Germany). The qPCR conditions were as follows: 15 s 95 °C, 20 s 58 °C, 25 s 72 °C (40 cycles), followed by melt curve analysis. All qPCR reactions were performed using two biological replicates and two technical replicates. For these replicates, plants were grown and treated under identical conditions as for transcriptome analysis. Fold change was calculated using the delta CT method with FRLP as a control gene for 7 dpi samples and Actin2 as a control gene for 14 dpi samples [29]. Primer sequences for the genes, which were verified through qPCR, are presented in Supplemental Table S4.

## 3. Results

### 3.1. Virus Infection of RNA Source Plants

All plants inoculated by each TEV strain (HAT, Mex21, and N) became infected, and their respective type of symptom development occurred as described previously [13]. Systemic vein-clearing developed for all virus-inoculated plants (for each of the three TEV strains) by 7 dpi. At 10 dpi, early stages of systemic mosaic symptoms were apparent with the distinct strain-specific symptoms [13] occurring by 14 dpi.

**Figure 1:**
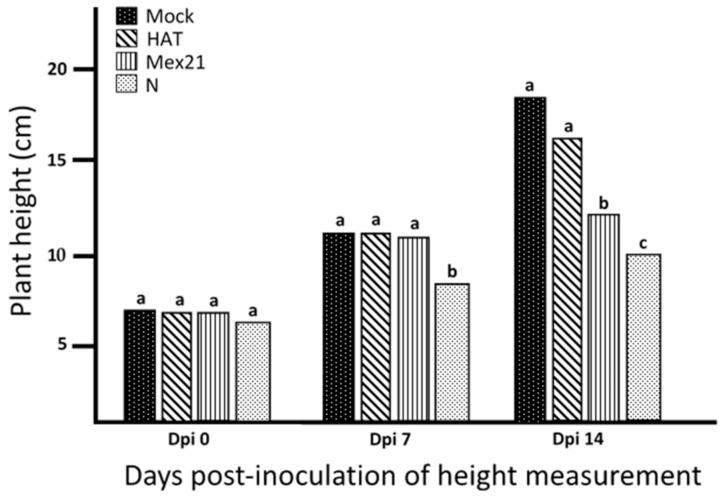
Height of plants infected with each of three *Tobacco etch virus* (TEV) strains. Mean value of height (cm) measured from soil-line to apical bud on the main stem of plants infected with TEV strain HAT, Mex21 or N along with buffer-mock control (Mock), measured at 0 (day of inoculation), 7 and 14 days post-inoculation (dpi). Statistical comparisons were made within a sample date; treatments followed by the same letter are not significantly different (Glimmix by Tukey–Kramer with *p* = 0.05).

Plant heights, a measure from soil-line to apical tip along the main stem, did not differ among treatments at the day of inoculation (dpi 0) (Figure 1). By 7 dpi, N-infected plants were significantly shorter than plants in the other treatments, whereas no difference in plant height occurred among buffer-mock control, HAT- and Mex21-infected plants. At 14 dpi, buffer-mock control and HAT infected plants did not differ significantly in height; however, Mex21-infected plants were significantly shorter than HAT-infected and buffer-mock control plants and, N-infected plants were significantly shorter than plants in each of the other treatments (Figure 1).

**Figure 2:**
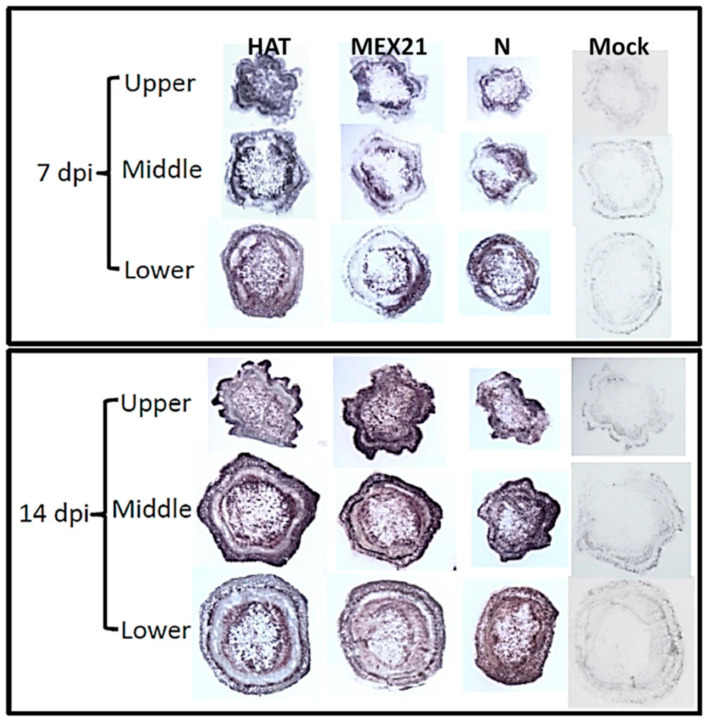
Accumulation of *Tobacco etch virus* (TEV) strains HAT, Mex21 or N in stem segments of Calwonder pepper plants. Virus accumulation was determined by immuno-tissue blot analysis at 7 and 14 days post-inoculation (dpi). Stem segments include the internode below inoculated leaves (lower), immediately above inoculated leaves (middle) and an upper internode close to the apical bud. The antibody-antigen (viral coat protein) reaction is detected by development of a reddish/brown stain. Buffer-mock control plants (Mock) were examined in a similar manner as viral treated samples and show no stain indicating no viral presence.

The extent of infection within plant stems was evaluated for each TEV strain using immuno-tissue blot analysis (Figure 2). This procedure detects viral coat protein with an antibody-antigen (coat protein) reaction that leads to a dark red/brown staining of stem prints. Stem prints were generated at 7 and 14 dpi for HAT, Mex21, N infected plants as well as the buffer-mock control. There is extensive immuno-staining throughout stem segments for each TEV strain at 7 and 14 dpi (Figure 2). These data illustrate the rapid spread of each TEV strain throughout each plant one week after inoculation and, of particular importance, detection of virus in the upper portions of the stem where RNA samples would be taken for transcriptome analysis. No immuno-staining occurred on tissue prints from buffer-mock control plants.

### 3.2. Transcriptome of TEV Strain-Infected and Buffer-Mock Control Plants

In order to understand changes in gene expression levels in pepper plants infected by each TEV strain, RNA sequencing was carried out on total RNAs isolated from the apical portion of the main stem of infected and buffer-mock control plants. Total RNAs were used to generate cDNA libraries from which single-end Illumina GAIIX RNA-sequencing was performed. This generated a total of 333,589,860 1 × 54 bp reads from all samples and, on average, 20.8 million reads per sample, which were then aligned to the *C. annuum* reference genome. The number of uniquely aligned reads was on average 16.5 million or 79% from all 7 dpi samples and 14.5 million or 70% from all 14 dpi samples from which gene expression was quantified, using the total number of reads per sample that uniquely aligned to the reference genome binned by gene (Table 1). Genes used for differential expression (DE) analysis were restricted to those found to be significantly regulated based on a *p*_adj_ < 0.05 (Tables S1 and S2) as compared among samples.

A further analysis of RNA sequencing alignment efficiency revealed that decreased percent alignments were found only in TEV infected samples and were more pronounced with increasing TEV stain-related disease severity. The average percent of uniquely mapping reads for 7 dpi vs. 14 dpi for the buffer-mock control is 86.3 vs. 86.0; HAT 83.2 vs. 84.0; Mex21 83.6 vs. 72.8; and N 80.3 vs. 53.2 (Table 1). All sequencing reads for each plant sample were re-examined to include potential alignment to the TEV genome sequence of each respective infected strain [12]. The change in level of RNA sequencing read alignment was found to be largely due to sequencing of the TEV RNA genome, instead of isolated plant RNA, roughly in response to TEV infection. For the mildest disease-inducing TEV strain, HAT, there is a 2–3% average read alignment to the viral genome with no increases from 7 to 14 dpi. In contrast, the more severe disease-inducing TEV strains both showed a large increase in average percent TEV read alignment from 7 to 14 dpi: for Mex21 from 3.1 to 15.5, and for N from 4.1 to 13.8 (Table 2). This appears to correspond to the level of disease severity and impact on pepper plant growth (e.g., height) (Figure 1 and Figure 2); however, it does not reflect levels of virus accumulation in foliar tissues.

**Figure 3:**
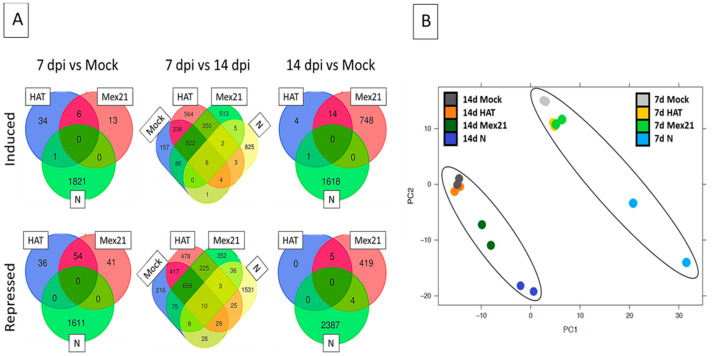
Transcriptome analysis reveals distinct patterns of *Tobacco etch virus* (TEV) gene regulation. (**A**) Venn diagrams indicating the numbers of pepper differentially expressed genes (DEGs) significantly regulated (induced or repressed) by TEV strains HAT, Mex21 or N at 7 or 14 days post-inoculation (dpi) compared to buffer-mock control plants (Mock) or between the same treatment sample at 7 and 14 dpi from RNA-sequencing analysis. Sample treatments are labeled and represented by distinct colors in each diagram using only significantly regulated DEGs with *p*_adj_ < 0.05. (**B**) Principle components analysis (PCA) of each RNA-sequencing transcript replicate, depicted as ovals, indicates a clustering of samples corresponding to days post-inoculation of sample collection. Changes within each oval appear to coincide with severity of TEV strain compared to buffer-mock control samples as a mix of PC1 and PC2.

The transcriptomic data were then used to generate three separate comparisons of DEGs (Figure 3). First, at 7 dpi, transcriptomic datum for each of the TEV strains was compared to the buffer-mock control sample as an examination of the earlier stage of TEV-induced symptoms. Similar comparisons were made at 14 dpi, a time point when disease symptoms and impacts on plant growth were clearly visible and distinguishable among strains. Finally, effects on transcript expression within each treatment (HAT to HAT, Mex21 to Mex21, N to N, and buffer-mock control to buffer-mock control) were compared between the 7 and 14 dpi stages of disease development. A Principal Component Analysis (PCA) and variance decomposition (both as implemented in SAS JMP Genomics 5.1) of the overall, full transcriptome dataset (*n* = 16) were made, indicating close grouping of replicates, but also showing distinct differences between samples at 7 and 14 dpi as indicated by ovals in Figure 3B. The 7 dpi sample replicates of the buffer-mock control treatment are clustered but distinct from both HAT and Mex21 samples, while N samples are further separated. The 14 dpi samples of buffer-mock control and HAT are clustered, with Mex21 and N further and individually separated along a diagonal axis (Figure 3B).

The number of significant DEGs was examined, made at the initial stage of symptom development (7 dpi) by comparing transcriptomes from TEV infected plants versus those from buffer-mock control plants of the same age. Only a small number of DEGs were found for HAT (131) and Mex21 (114) infected plants at this early stage in disease development (Figure 3; Tables S1 and S2). However, N-infected plants had many DEGs identified (3433), with 1822 induced and 1611 repressed (Figure 3; Tables S1 and S2). A similar examination of the 7 dpi lists of DEGs for connections to disease response or being disease-related showed no genes of this type in the HAT list, 2 in the Mex21 list, and 62 in the N list (Table S3).

Genes identified on this list as regulated by these treatments consist of several different general disease related genes including Leucine Rich Repeats (LRRs) and genes involved in resistance to *Tobacco mosaic virus* (TMV), nematode, and blight (Table S3). A general comparison of common significant DEGs among TEV-infected plants revealed no overlapping genes regulated in the same manner between HAT, Mex21, and N versus the buffer-mock control treatment. There was also little overlap between any N-related DEGs and either HAT- or Mex21-related DEGs, although there were several common HAT and Mex21 DEGs. These results reflect the initial stages of disease development among the TEV strains where disease-induced stunting occurred for N-infected plants but not for plants infected with HAT or Mex21 (Figure 1). The large number of DEGs in N-infected plants suggests a more advanced state of infection, although since the HAT and Mex21 infected plants did not reach a similarly high level of genes affected, it may indicate infection of plants by N results in a distinctly different impact on the plant leading to greater changes in significant DEGs. The approximate 50% overlap of DEGs between HAT- and Mex21-infected plants indicates a similar set of symptom-related gene response at this (7 dpi) stage of infection (Figure 3).

By 14 dpi, the distinct TEV strain-specific systemic symptoms were clearly observed (Figure 1), and these corresponded to a similar pattern of increased numbers of DEGs relative to 7 dpi (Figure 3). The smallest number of DEGs (24) was for HAT versus the buffer-mock control, suggesting only minor changes in gene expression occurred for the mildest disease inducing TEV strain. A moderate number of DEGs was identified for Mex21 versus the buffer-mock control (1190), with 762 induced and 428 repressed (Figure 3). Finally, the largest number of DEGs occurred for N versus the buffer-mock control (4010), with 1619 induced and 2391 repressed (Figure 3). This pattern of increasing numbers of DEGs with increasing TEV strain-related symptom severity suggests that the infection may largely function at a transcriptional level to influence plant disease phenotype. Interestingly, a comparison of similar DEGs, as shown in the Venn diagrams, reveals a distinctly different pattern of gene expression between Mex21- and N-infected plants. HAT-infected plants have very few DEGs, most of which, however, overlap with Mex21 DEGs. There are very few DEGs that overlap between Mex21 and N treatments and no common DEGs occur among all three strains of TEV-infected plants. An examination of DEGs at 14 dpi reveals no annotated disease-related genes for HAT, 10 repressed genes in for Mex21, and 7 induced genes in for N (Table S3). It is unclear how this directly relates to the total number of DEGs, except that this manual examination likely underestimates the number of genes related to disease response based on incomplete annotation of the pepper genome at present.

Finally, transcriptomic values were compared between 7 and 14 dpi using the lists of significant DEGs (Figure 3). For buffer-mock control plants, 2762 DEGs were identified as those expected to occur during this period in response to increased plant age and developmental changes. There were roughly similar numbers of DEGs induced (1321) and repressed (1441). Comparable levels of DEGs were found for the 7 to 14 dpi comparison within TEV infected samples: 3832 for HAT, 3151 for Mex21, and 2516 for N. In the HAT and Mex21 DEG lists, similar to the list for buffer-mock control samples, there were nearly equal numbers of induced and repressed genes. This was not the case for N sample DEGs, where there were nearly twice as many repressed genes as there were induced genes. Although there were large numbers of DEGs for each of the TEV strain infected plants, upon subtraction of the buffer-mock control DEGs that overlapped with the TEV strain lists, a much smaller number of TEV-infection related DEGs was identified. The numbers of DEGs from TEV-infected plants (buffer-mock control list subtracted) for the 7 to 14 dpi comparison were: 1042 for HAT, 867 for Mex21, and 2356 for N (Figure 3A). Approximately half of HAT and Mex21 DEGs overlap, whereas there were few overlaps with N DEGs, similar to 7 and 14 dpi individual comparisons. Only five DEGs were regulated in a similar manner among the TEV strains, which were not also significantly regulated in the buffer-mock control samples. Most of the TEV strain-related DEGs identified in these 7 to 14 dpi evaluations were unique for HAT (63%), Mex21 (58%), and N (97%) infected plants (Figure 3). These findings of largely distinct DEG profiles were similarly borne out by examining the list of disease-related DEGs for the 7 to 14 dpi comparisons (Table 3). Interestingly, several disease-related genes were significantly regulated even in the buffer-mock control treatment 7 to14 dpi comparison, presumably reflecting normal plant developmental changes in defense response. For each TEV strain treatment comparison, approximately 50% of disease-related genes were unique compared with those from the buffer-mock control samples. Despite this difference, there was little overlap of genes regulated in the same manner among the TEV strains, suggesting that each TEV strain induces distinct disease-related gene expression in pepper plants (Table 3). Perhaps, the most interesting overlap of DEGs from these comparisons came from five genes: two late blight resistance R1B-16-like (CA11g18010, CA09g10460), a NBS-LRR resistance (CA07g12630), a natural resistance-associated macrophage (CA04g18210), and a disease resistance BS2 protein (CA09g17400). Each of these genes were significantly regulated in a similar direction with comparable log2 FC levels in the buffer-mock control, HAT, and Mex21 samples, whereas being significantly regulated in the opposite direction in N samples (Table 3). Both late blight resistance genes and the NBS-LRR resistance gene are induced in the buffer-mock control, HAT, and Mex21 plants, but repressed in N-infected plants, with the converse being true for the other two genes (Table 3). This clearly indicates a unique gene regulation pattern in response to TEV-N infection.

qPCR was performed in order to confirm the results of RNA-sequencing on six DEGs affected by TEV infection from 7 to 14 dpi (Table 4). TEV-N infected samples versus buffer-mock control samples were examined as these showed some of the most pronounced changes. This was done by comparing expression levels of genes with N versus buffer-mock control samples from 7 dpi and then N versus buffer-mock control samples from 14 dpi, which were then further examined for differences in fold change between these individually controlled samples. Results showed similar expression directionality and regulation as a decrease in expression from 7 to 14 dpi for N infected samples for the genes examined indicating that the gene expression values obtained from RNA-sequencing were accurate.

An analysis of gene ontology or GO terms was performed for each list of DEGs at 7 dpi, 14 dpi, and from the comparison of these treatment times. This resulted in the identification of several expected GO categories in abundance including: for Biological Processes, “Response to Stimulus”, “Developmental Process”, “Multi-organism Process” and “Immune System Process”; for Molecular Function, “Catalytic Activity” and “Antioxidant Activity” (Figures S1–S3). Although many individual genes were changed, little change was noted between any of the general categories of DEGs, suggesting that no clearly identifiable process was uniquely altered for any of the treatments examined. It is also possible that a lack of strong GO term annotation for the pepper genome may make it difficult to fully detect differences.

## 4. Discussion

TEV causes detrimental reduction in crop yields across members of the family *Solanaceae* including bell pepper, *C. annuum*. The three TEV strains used in this study, although closely related genetically, are distinctly different in their pathological properties [12,13]. While the virus genetics and disease impacts on pepper plant growth and development have been described for each TEV strain, it is essential to begin dissection of changes in plant gene expression in response to TEV infection and disease progress. In this study, genome-wide changes in gene expression were examined for the apical stem region of pepper plants at an early (7 dpi) and a later (14 dpi) stage of disease development. Although disease symptoms and growth impacts on pepper plants have been well described for each TEV strain, three factors were measured during this current study as indicators for potential effects on gene expression in the apical segment of the stem: systemic symptom expression in foliar tissues, accumulation of virus in stem segments using immuno-tissue blot analysis and plant height as a measure of stem length. The initial systemic symptom, vein-clearing, occurred for each TEV strain by 7 dpi, development of foliar mosaic symptoms by 10 dpi and the distinct TEV strain-specific systemic symptoms by 14 dpi. Each TEV strain was detected in all tested segments along the stem by immuno-tissue blot analysis by 7 dpi. Negative impacts on plant height occurred for N-infected plants by 7 dpi and for Mex21- and N-infected plants by 14 dpi. These data clearly indicate widespread occurrence of each TEV strain within the plant by 7 dpi and viral strain-related impacts on plant growth from 7 to 14 dpi. Therefore, impacts on pepper plant transcripts, relative to the buffer-mock control and among TEV strain treatments, were anticipated from the transcriptomes. It is interesting to consider the nature of N’s impact on the apical stem since stunting occurred early in systemic infection. Each of the TEV strains moved to the apical region at a relatively similar time, based on the immuno-tissue blots, but infection by N led to greater effects on growth and gene expression/suppression. Microscopy studies to determine virus location, e.g., does N invade the apical meristem, whereas HAT and Mex21 do not, and cytopathological effects on the apical meristem might aid to explain the different impacts among TEV strains on pepper plant growth. Although indirectly related, does N have a greater capacity to suppress gene silencing thereby leading to a more severe disease than caused by HAT and Mex21? This latter consideration is complicated by the fact that N does not accumulate (at least in leaf tissues) to as high a level as HAT.

The distinct difference observed for N-infected plants when compared with HAT- and Mex21-infected plants is interesting considering that at 7 dpi, expression of foliar symptoms did not differ among the virus treatments, although impacts on growth were already occurring for N-infected plants (Figure 1). The DEGs, however, clearly distinguish impacts of N infection on pepper plants from those of HAT and Mex21. Interestingly, 60 of the 62 disease-related DEGs from 7 dpi N-infected plants were induced, suggesting the pepper plant is in the process of responding to the infection (Table S3). 

The percentage of reads from RNA sequencing at 7 dpi were similar among the TEV strains for alignment to the pepper genome and the respective TEV strain (Table 2). For samples from HAT-infected plants, the percentage of reads remained similar at 7 and 14 dpi. However, for both Mex21- and N-infected plant samples, the percentage of reads aligned to the pepper genome declined from 7 to 14 dpi, whereas the percentage of reads that aligned to the respective viral strain genome increased during that same time frame. At 14 dpi, plants were expressing the distinct systemic symptoms for each TEV strain with the decline in percentage of aligned reads to the pepper genome corresponding to the increase in disease severity induced by the TEV strain. These results could suggest with an increase in disease severity for these TEV strains, there is a corresponding decline in the pepper gene expression in the apical bud region of the stem. For example, the apical portions of HAT-infected plants are only mildly affected by virus and appear similar to comparable tissues of buffer-mock control plants. In contrast, the apical region of N-infected plants is extremely chlorotic and with a nearly complete halting of growth. This apparent “shut down” in N-infected plants might be the result of reduced maintenance of the apical bud and suggests with an increase in disease severity for these TEV strains, there is a corresponding decline in the pepper transcript alignments representing gene expression levels.

Examination of the number of DEGs comparing individual TEV strains versus the buffer-mock control at 7 dpi revealed a small number of genes were significantly affected in HAT- and Mex21-infected samples, whereas several thousand genes were regulated in N-infected samples (Figure 3). At 14 dpi, HAT-infected samples continued to have a small number of DEGs, but DEG numbers increased to ~1200 and 4000 for Mex21- and N-infected samples, respectively (Figure 3). These results strongly parallel the severity of TEV strain-specific symptoms and impacts on pepper plant growth seen here (Figure 1 and Figure 2) and previously [13].

An additional analysis was made within each treatment comparing data from 7 and 14 dpi in order to identify genes affected during different stages in infection and disease development. There was no overlap of DEGs within individual TEV strain treatments at 7 or 14 dpi and few overlapping DEGs comparing 7 to 14 dpi (Figure 3). A few DEGs that overlapped from 7 to 14 dpi were further examined by qPCR and showed similar levels and direction of expression, validating RNA sequencing results (Table 4). Several of these overlapping genes are involved in auxin, cytokinin, and ethylene processes linking these hormones to altered growth patterns during viral infection (Table 4). Samples from the buffer-mock control treatment had similar numbers of DEGs when comparing 7 to 14 dpi; these DEGs are likely involved in changes in standard plant developmental processes as the plant ages and most do not overlap with DEGs from viral infected samples (Figure 3). These overall patterns of changes in DEGs at 7 and 14 dpi between TEV strains and plant age can also be seen in the PCA results where differences appear to coincide with TEV strain-related symptom severity compared to buffer-mock control at each time point (within each oval, Figure 3B). Variation between samples appears to correspond to both developmental timing (dpi) and disease severity, as a mix of PC1 and PC2 (Figure 3B). Taken together, this suggests a combination of plant development and viral infection influencing patterns of gene regulation.

Together, these data show that each TEV strain affects patterns of gene expression in the host pepper plant distinctly, at least during these earlier stages of disease progress within the plant. Although these DEG profiles for each TEV strain appear to be distinct, similarities exist upon examination of each group of genes based on their function as determined by GO enrichment analysis (Tables S1–S3). An example is disease-related DEGs (Table 3) where few or no DEGs were identified at 7 dpi for HAT- and Mex21-infected samples, and 60 DEGs were induced for N-infected samples. There were no disease related DEGs at 14 dpi for HAT-infected samples and similar, yet, non-overlapping numbers for Mex21- and N-infected samples. From 7 to 14 dpi comparisons, similar numbers were found for each TEV strain and buffer-mock control samples, again with little or no overlap. These comparisons indicate that while different sets of disease response and resistance genes were affected in each grouping, the plant was responding to the infection with an overall similar group of genes. It is interesting that there appears to be a set of disease-related genes that are standardly regulated in buffer-mock plants, as seen from buffer-mock control samples, which changes as the plant ages during its development. One set of disease-related DEGs that did show overlap in the 7 to 14 dpi comparisons were two late blight resistance R1B16-like genes (CA11g18010 and CA09g10460). The expression of these genes closely parallels the level of disease symptom severity seen during TEV infection in a strain-specific manner: both genes show high induction during no infection (buffer-mock control), mild disease (HAT), and moderate disease (Mex21), and high levels of repression during severe disease (N) (Table 3). A similar pattern was found for a NBS-LRR resistance gene (CA07g12630). In direct contrast was the repression of both a natural resistance-associated macrophage (CA04g18210) and a disease resistance BS2 protein (CA09g17400) in buffer-mock control, HAT, and Mex21 samples, whereas both genes were induced in N-infected samples (Table 3). These blight and LRR resistance genes are common DEGs that are found in viral responses and in previous plant transcriptome experiments [18]. Together these findings indicate a potential role for each of these five genes in the pepper plant response to TEV infection. In addition, several of the DEGs selected for verification also appear to have potentially interesting roles in the regulation of either viral infection (e.g., the TMV-induced CaTin1) or in hormone-based plant growth such as a cytokinin-related gene, CaCRF1, and an auxin-induced gene, AIP (Table 4). CaTin1 was found in previous studies of pepper treated with TMV as an early induced viral response gene [30]. The identification of CaTin1 at a high level in this study in samples taken at 7 dpi, but reduced by 14 dpi, could suggest an associated plant-viral interaction during infection. Perhaps viral-related in response, but TEV and TMV are not related viruses suggesting CaTin1 as a broader response (Table 4). Some of the breadth of CaTIN1 in stress response has been shown previously from its regulation from H_2_O_2_ and ethylene, although not salicylic acid [31]. Thus, CaTin1 could be an interesting gene for future biotic and abiotic studies. Cytokinin Response Factors, such as CaCRF1, have been linked primarily to cytokinin signaling responses, but have also been connected to several different abiotic stresses [32]. Although there has been little work of CRFs or other cytokinin-related genes to viral infection [33], the findings here suggest that maybe this should be further investigated. The auxin induced AIP gene is interesting as auxin was shown to be connected to TMV-infected tobacco plants and a potential reprograming of plants to allow greater viral movement [34].

Overall, it is generally difficult to fully compare the transcriptomic results of this study with those of prior studies as there are unfortunately only a limited number of transcriptome level analyses of viral-treated plants and even fewer with pepper [18]. Prior transcriptome-based studies were clearly hindered before completion of the *C. annuum* genome sequence in 2014, which allowed simpler assembly of RNA sequence reads to a well-established genome [14]. Of the transcriptome studies that have been completed in pepper, most have focused primarily on fruit development or characteristics, capsaicin production or chilling stress [14,15,16,17]. There have been relatively few transcriptomic studies focused on virus infected pepper plants [18]. An early study examined PGMV-infected plants via 454 pyrosequencing at 9 dpi [35], while a more recent study focused on pepper plants infected with CMV during the early stages of infection (from 6 h to 72 h) in leaf tissues using Illumina-based as well as SMRT-based RNA sequencing technologies [36]. Each of these studies revealed general sets of DEGs that were connected to stress and disease response as well as hormones, such as ethylene and auxin. All of these were found in this study, as discussed above for disease and auxin, but also for the ethylene-related gene, ACO (Tables S1 and S2). There are few transcriptome studies involving TEV-infected plants [18]. Agudelo-Romero et al. [19] and Hillung et al. [20] investigated gene expression profiles of different *Arabidopsis thaliana* ecotypes infected by TEV strain *At*17b. Microarray analysis revealed TEV impacted a wide array of Arabidopsis genes, especially those associated with stress response pathways [19]. They observed a larger overall response in Arabidopsis gene expression to TEV infection, representing more of a global response than one specific to TEV. High-throughput transcript profiling allowed classification of Arabidopsis ecotypes based on their gene expression patterns in response to TEV-*At*17b infection [20]. Gene expression corresponded to disease severity induced by TEV-*At*17b whereby severely affected Arabidopsis ecotypes had up-regulation of defense-associated genes, but ecotypes with milder or tolerant responses to TEV-*At*17b infection up-regulated genes associated with abiotic stresses.

## Figures and Tables

**Table 1:** Total reads from RNA-sequencing runs.

Samples ^a^	Reads Per	% Reads Uniquely	Reads	% Reads Uniquely	Reads
	Sample ^b^	Aligned	Aligned ^b^	Aligned ^b^	Aligned
7 dpi Mock (1)	18,974,511	16,346,906	86.20	15,529,610	81.80
7 dpi Mock (2)	20,301,364	17,521,361	86.30	16,644,069	82.00
7 dpi HAT (1)	20,919,036	17,346,241	82.90	16,473,194	78.70
7 dpi HAT (2)	21,866,966	18,241,859	83.40	17,324,729	79.20
7 dpi Mex21 (1)	21,902,531	18,325,050	83.70	17,366,834	79.30
7 dpi Mex21 (2)	22,172,613	18,505,468	83.50	17,558,247	79.20
7 dpi N (1)	19,018,389	15,053,277	79.20	14,224,515	74.80
7 dpi N (2)	21,561,491	17,528,288	81.30	16,547,524	76.70
14 dpi Mock (1)	20,439,241	17,553,946	85.90	16,649,649	81.50
14 dpi Mock (2)	19,268,355	16,568,352	86.00	15,696,565	81.50
14 dpi HAT (1)	21,163,737	17,695,058	83.60	16,761,885	79.20
14 dpi HAT (2)	20,397,503	17,195,270	84.30	16,288,450	79.90
14 dpi Mex21 (1)	19,084,583	13,684,022	71.70	12,955,283	67.90
14 dpi Mex21 (2)	22,247,287	16,450,047	73.90	15,541,636	69.90
14 dpi N (1)	23,209,601	11,424,127	49.20	10,768,080	46.40
14 dpi N (2)	21,062,652	12,048,713	57.20	11,358,940	53.90

^a^ Samples are of *Capsicum annuum* cv. Calwonder plant treatments buffer-mock control (Mock) or *Tobacco etch virus* strains HAT, Mex21, or N after 7 or 14 days post-inoculation (dpi). ^b^ The total number and percent of RNA-sequencing reads from each biological replicate as well as the number and percent of those reads that were then uniquely aligned to the *C. annuum* cv CM334 pepper reference genome.

**Table 2:** Percent reads from RNA-sequencing runs aligned to the *Capsicum annuum* genome versus the *Tobacco etch virus* (TEV) genome.

Samples ^a^	% Reads	% Reads	Samples ^a^	% Reads	% Reads	Samples ^a^	% Reads	% Reads
	Aligned to *C. annuum* ^b^	Aligned to TEV-HAT ^b^		Aligned to *C. annuum* ^b^	Aligned to TEV-Mex21 ^b^		Aligned to *C. annuum* ^b^	Aligned to TEV-N ^b^
7 dpi HAT (1)	82.90	3.79	7 dpi Mex (1)	83.70	3.08	7 dpi N (1)	79.20	4.75
7 dpi HAT (2)	83.40	3.42	7 dpi Mex (2)	83.50	3.16	7 dpi N (2)	81.30	3.45
14 dpi HAT (1)	83.60	2.89	14 dpi Mex (1)	71.70	16.67	14 dpi N (1)	49.20	15.71
14 dpi HAT (2)	84.30	2.19	14 dpi Mex (2)	73.90	14.28	14 dpi N (2)	57.20	11.79

^a^ Samples are of *C. annuum* cv. Calwonder plants infected with TEV strains HAT, Mex21, or N after 7 or 14 days post-inoculation (dpi). ^b^ The percent of total RNA-sequencing reads from each biological replicate that were aligned to the *C*. *annuum* cv CM334 pepper reference genome or the TEV-strain specific genome (HAT-AU, Mex21, or N).

**Table 3:** *Tobacco etch virus* (TEV) strain-specific disease-related differentially expressed genes (DEGs) in *Capsicum annuum* plants from 7 to 14 days post-inoculation (dpi).

Comparison 7 dpi vs. 14 dpi Buffer-Mock Control ^a^			
Gene ID ^b^	Gene Description	log2 FC	*p* adj
CA11g18010	Late blight resistance protein homolog R1B-16-like	2.16	2.32 × 10^−6^
CA09g10460	Late blight resistance protein homolog R1B-16-like	1.85	2.62 × 10^−7^
CA06g15650	Nematode resistance-like protein	1.64	0.02524
CA10g12800	Disease resistance protein	0.93	0.0059
CA12g02350	Disease resistance protein A19 (Fragment)	0.91	0.04575
CA11g05830	Potyviral capsid protein interacting protein 1	0.84	8.39 × 10^−6^
CA07g12630	NBS-LRR type disease resistance protein	0.67	0.00018
CA06g01130	NBS-LRR resistance protein-like protein	0.6	0.0273
CA06g19920	TMV resistance protein N-like	0.55	0.00155
CA02g19570	Nematode resistance-like protein	0.5	0.02059
CA03g03390	Late blight resistance protein homolog R1B-23-like	0.4	0.0327
CA02g24750	Root-knot nematode resistance protein	−0.39	0.04397
CA12g20500	Disease resistance protein	−0.53	0.00814
CA04g18210	Natural resistance-associated macrophage protein	−0.74	0.00032
CA09g17400	Disease resistance protein BS2	−0.82	0.0006
CA06g10690	Disease resistance response protein 206-like	−0.96	0.00966
CA04g17660	Disease resistance protein RPP13-like	−0.96	0.01513
CA06g02450	NBS-LRR resistance protein-like protein	−1.04	2.99 × 10^−5^
CA09g12200	Verticillium wilt disease resistance protein	−1.41	9.25 × 10^−7^
Comparison 7 dpi vs. 14 dpi HAT ^a^			
Gene ID ^b^	Gene Description	log2 FC	*p* adj
CA11g18010	Late blight resistance protein homolog R1B-16-like	1.84	0.0044
CA09g10460	Late blight resistance protein homolog R1B-16-like	1.56	7.49 × 10^−11^
CA09g00920	Disease resistance protein BS2	1.17	0.00069
CA10g19890	Disease resistance RPP13-like protein 4-like	1.07	0.04508
CA01g01370	Grave disease carrier protein, putative	0.92	0.00255
CA10g12800	Disease resistance protein 1	0.7	0.0422
CA11g05830	Potyviral capsid protein interacting protein	0.63	0.00039
CA07g12630	NBS-LRR type disease resistance protein	0.48	0.00069
CA01g08330	Blight resistance protein	0.43	0.03268
CA11g02410	Late blight resistance protein homolog R1C-3-like	0.3	0.0387
CA02g18130	Tobamovirus multiplication 1	−0.43	0.01041
CA04g20220	Xenotropic and polytropic retrovirus receptor	−0.47	0.00935
CA02g11830	Nbs-lrr resistance protein	−0.56	0.01068
CA09g17400	Disease resistance protein BS2	−0.57	0.02554
CA01g17390	Nbs-lrr resistance protein	−0.59	0.00276
CA05g00030	Cc-nbs-lrr resistance protein	−0.69	0.01179
CA04g18210	Natural resistance-associated macrophage protein	−0.78	4.73 × 10^−5^
CA06g01230	Late blight resistance protein Rpi-blb2	−0.81	0.01808
CA07g01130	Disease resistance protein BS2	−0.92	0.00587
CA06g03690	Root-knot nematode resistance protein	−1.38	0.02771
CA12g20430	Disease resistance protein	−1.55	0.00506
CA06g03680	Late blight resistance protein homolog R1B-14-like	−2.2	9.98 × 10^−5^
CA07g01000	NBS-coding resistance gene analog (Fragment)	−2.23	0.01325
CA09g18620	Nematode resistance-like protein	−3.68	0.04036
Comparison 7 dpi vs. 14 dpi Mex21 ^a^			
Gene ID ^b^	Gene Description	log2 FC	*p* adj
CA11g18010	Late blight resistance protein homolog R1B-16-like	1.66	0.00027
CA09g10460	Late blight resistance protein homolog R1B-16-like	1.29	3.39 × 10^−6^
CA06g05010	Antiviral helicase SKI2-like	1.23	0.02099
CA10g19760	NBS resistance protein	0.87	0.00401
CA06g12190	Late blight resistance protein homolog R1C-3-like	0.86	0.02072
CA07g12630	NBS-LRR type disease resistance protein	0.62	0.00026
CA11g05830	Potyviral capsid protein interacting protein 1	0.54	0.01863
CA03g03390	Late blight resistance protein homolog R1B-23-like	0.5	0.00761
CA11g02410	Late blight resistance protein homolog R1C-3-like	0.49	0.00338
CA02g24750	Root-knot nematode resistance protein	−0.53	0.00412
CA09g17010	BED finger-nbs-lrr resistance protein	−0.56	0.04580
CA02g11830	Nbs-lrr resistance protein	−0.57	0.04129
CA04g18210	Natural resistance-associated macrophage protein	−0.71	0.00133
CA05g00030	Cc-nbs-lrr resistance protein	−0.72	0.02653
CA05g17790	Late blight resistance protein homolog R1C-3-like	−0.74	0.03569
CA10g19860	Late blight resistance protein homolog R1B-17-like	−0.78	0.01313
CA06g02730	Root-knot nematode resistance protein	−0.84	0.0245
CA04g02910	Cc-nbs-lrr resistance protein, isoform 1	−0.84	0.04803
CA01g17390	Nbs-lrr resistance protein	−0.85	0.00013
CA11g06220	Tir-nbs-lrr resistance protein	−1.06	0.00748
CA07g00840	Disease resistance protein BS2	−1.16	0.04134
CA01g32390	Disease resistance protein At4g27190-like	−1.21	0.02812
CA09g17400	Disease resistance protein BS2	−1.24	5.25 × 10^−7^
CA06g02450	NBS-LRR resistance protein-like protein	−1.28	0.00087
CA12g20430	Disease resistance protein	−2.27	7.54 × 10^−5^
CA05g04310	Late blight resistance protein homolog R1A-10-like	−2.34	7.64 × 10^−5^
CA02g19860	Disease resistance response protein 206-like	−2.63	1.61 × 10^−6^
CA08g01440	Late blight resistance protein homolog R1A-10-like	−2.70	0.0118
Comparison 7 dpi vs. 14 dpi N ^a^			
Gene ID ^b^	Gene Description	log2 FC	*p* adj
CA09g16190	Disease resistance protein	1.64	0.02537
CA07g00840	Disease resistance protein BS2	1.62	0.00905
CA06g02060	Late blight resistance protein homolog R1B-14-like	1.60	0.00124
CA06g01750	Root-knot nematode resistance protein	1.49	0.01234
CA07g00860	Disease resistance protein BS2	1.33	0.02024
CA06g15080	Nematode resistance-like protein	1.29	0.04563
CA04g01190	Disease resistance protein Cf-2.1-like	1.14	0.02752
CA07g00880	Resistance protein PSH-RGH6	1.13	0.00427
CA11g06220	Tir-nbs-lrr resistance protein	1.09	0.02214
CA09g17400	Disease resistance protein BS2	0.76	0.04073
CA04g18210	Natural resistance-associated macrophage protein	0.71	0.04064
CA10g20630	Disease resistance protein At4g27220-like isoform X1	−0.71	0.02130
CA07g12630	NBS-LRR type disease resistance protein	−0.79	0.00043
CA10g19760	NBS resistance protein	−1.07	0.00772
CA09g10460	Late blight resistance protein homolog R1B-16-like	−1.13	0.00188
CA11g18690	Disease resistance RPP13-like protein 1-like isoform X2	−1.25	0.03543
CA12g06200	Resistance gene-like	−1.40	0.00075
CA02g19860	Disease resistance response protein 206-like	−1.56	0.00521
CA11g18010	Late blight resistance protein homolog R1B-16-like	−2.09	0.02585
CA12g16200	TMV resistance protein N-like	−2.40	0.03846

^a^ Significant DEGs within each treatment group from 7 to 14 dpi comparisons were examined by gene description for relation to disease response using the key words: disease, resistance, resistant, virus, and viral. Treatments included a buffer-mock control and TEV strains HAT, Mex21 and N. ^b^ Gene ID, description, log2-fold change (FC), and adjusted *p*-value (*p* adj) are given.

**Table 4:** qPCR confirmation of RNA sequencing transcriptomic results.

Samples ^a^	TIN1 ^b^	WRKY26 ^b^	FAD ^b^	CaCRF1 ^b^	AIP ^b^	PIP1 ^b^
	CA10g03990	CA02g14640	CA08g04180	CA06g25980	CA04g13560	Ca02g11250
N-RNAseq (7–14d)	−2.68	−2.08	−13.05	−3.16	−2.82	−5.46
N-qPCR (7–14d)	−2.74 ± 1.93	−1.47 ± 0.52	−9.73 ± 5.28	−2.31 ± 0.97	−1.74 ± 0.15	−4.15 ± 3.20
N-qPCR (7d)	5.27 ± 3.31	2.26 ± 0.90	11.67 ± 9.15	5.43 ± 1.68	2.24 ± 0.06	11.25 ± 5.43
N-qPCR (14d)	1.92 ± 0.43	1.54 ± 0.06	1.20 ± 0.09	2.35 ± 0.03	1.29 ± 0.25	2.71 ± 1.10

^a^ Individual comparisons of *Tobacco etch virus* (TEV-N) samples vs. buffer-mock control at 7 days post-inoculation (dpi) and 14 dpi by qPCR used to compute the 7d-14d comparison. ^b^ Six differentially expressed genes from comparisons of TEV- N-infected plants at 7 dpi vs. 14 dpi by RNA sequencing (top row) were selected for further verification of fold expression change by qPCR (second row). qPCR was performed using 2 biological replicates and 3 technical replicates treated in the same manner as for RNA-sequencing experiments and normalized to FRLP for 7 dpi and ACT2 for 14 dpi gene expression as controls. Gene ID and name are given.

## Data Availability

Raw sequence data are available for download at NCBI Sequence Read Archive under the BioProject ID: PRJNA476480, SRA accession: SRP150696.

## Supplementary Materials

The following are available online at https://www.mdpi.com/article/10.3390/v13050741/s1, Table S1 List of all induced DEGs from all comparisons—large excel file; Table S2 List of all repressed DEGs from all comparisons—large excel file; Table S3 List of disease-related DEGs; Table S4 List of real-time PCR primers used in this manuscript; Figure S1 Gene Ontology Term Analysis for 7 dpi samples; Figure S2 Gene Ontology Term Analysis for 7 vs. 14 dpi samples; Figure S3 Gene Ontology Term Analysis for 14 dpi samples.
