# Supplementary material for: Three Strains of Tobacco etch virus Distinctly Alter the Transcriptome of Apical Stem Tissue in Capsicum annuum during Infection
Source: Viruses. 2021 Apr 23;13(5):741. doi: 10.3390/v13050741 (PMC8145408; doi:10.3390/v13050741)
Supplement: Supplementary file 1 [file viruses-13-00741-s001.zip › Supplement figs and tables/Table S4 qPCR Primers (1).pptx]

## Slide 1
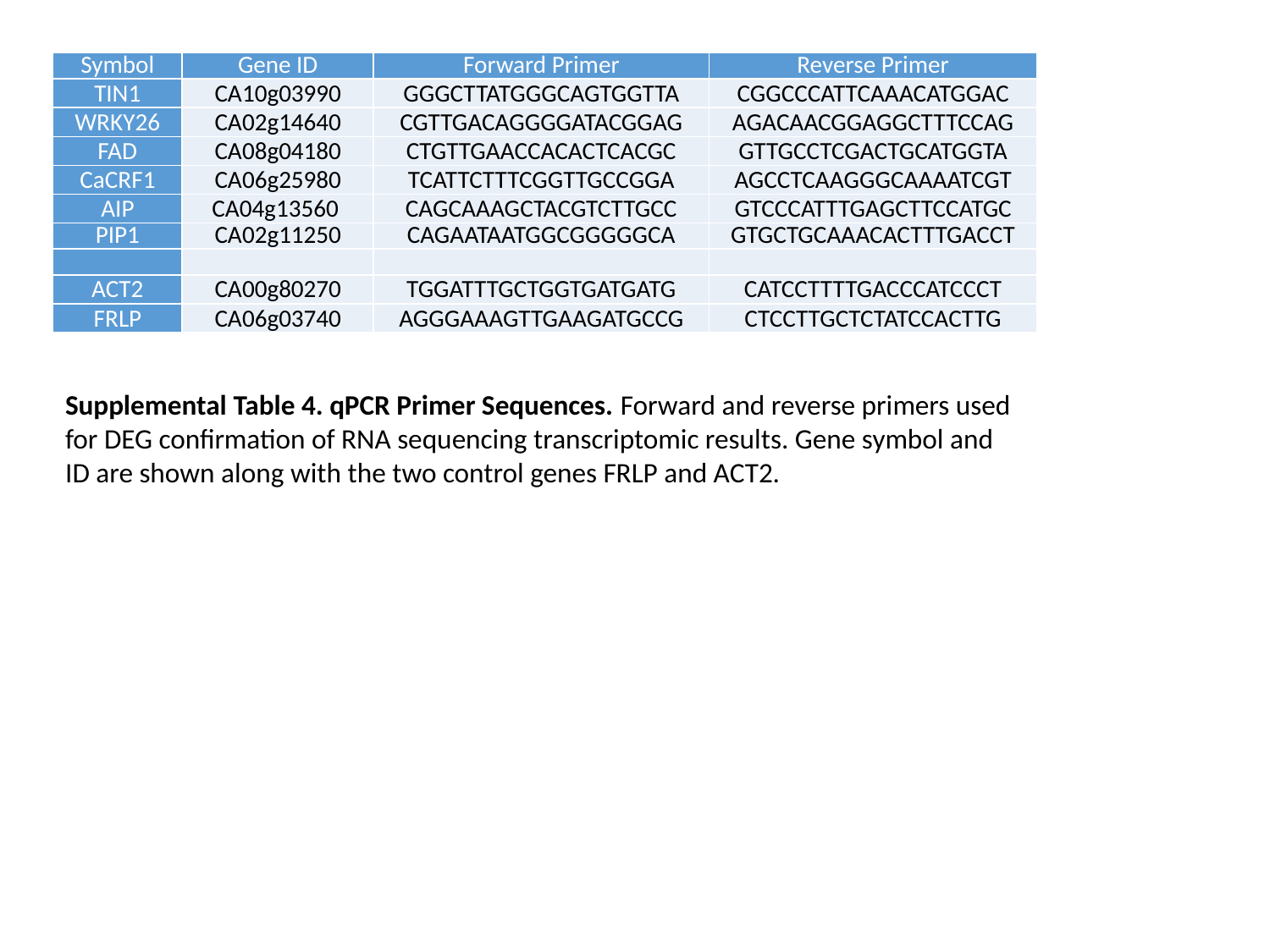

| Symbol | Gene ID | Forward Primer | Reverse Primer |
| --- | --- | --- | --- |
| TIN1 | CA10g03990 | GGGCTTATGGGCAGTGGTTA | CGGCCCATTCAAACATGGAC |
| WRKY26 | CA02g14640 | CGTTGACAGGGGATACGGAG | AGACAACGGAGGCTTTCCAG |
| FAD | CA08g04180 | CTGTTGAACCACACTCACGC | GTTGCCTCGACTGCATGGTA |
| CaCRF1 | CA06g25980 | TCATTCTTTCGGTTGCCGGA | AGCCTCAAGGGCAAAATCGT |
| AIP | CA04g13560 | CAGCAAAGCTACGTCTTGCC | GTCCCATTTGAGCTTCCATGC |
| PIP1 | CA02g11250 | CAGAATAATGGCGGGGGCA | GTGCTGCAAACACTTTGACCT |
| | | | |
| ACT2 | CA00g80270 | TGGATTTGCTGGTGATGATG | CATCCTTTTGACCCATCCCT |
| FRLP | CA06g03740 | AGGGAAAGTTGAAGATGCCG | CTCCTTGCTCTATCCACTTG |
Supplemental Table 4. qPCR Primer Sequences. Forward and reverse primers used for DEG confirmation of RNA sequencing transcriptomic results. Gene symbol and ID are shown along with the two control genes FRLP and ACT2.
