# Supplementary material for: Three Strains of Tobacco etch virus Distinctly Alter the Transcriptome of Apical Stem Tissue in Capsicum annuum during Infection
Source: Viruses. 2021 Apr 23;13(5):741. doi: 10.3390/v13050741 (PMC8145408; doi:10.3390/v13050741)
Supplement: Supplementary file 1 [file viruses-13-00741-s001.zip › Supplement figs and tables/Transcriptome supplemental fig. 3.pptx]

## Slide 1
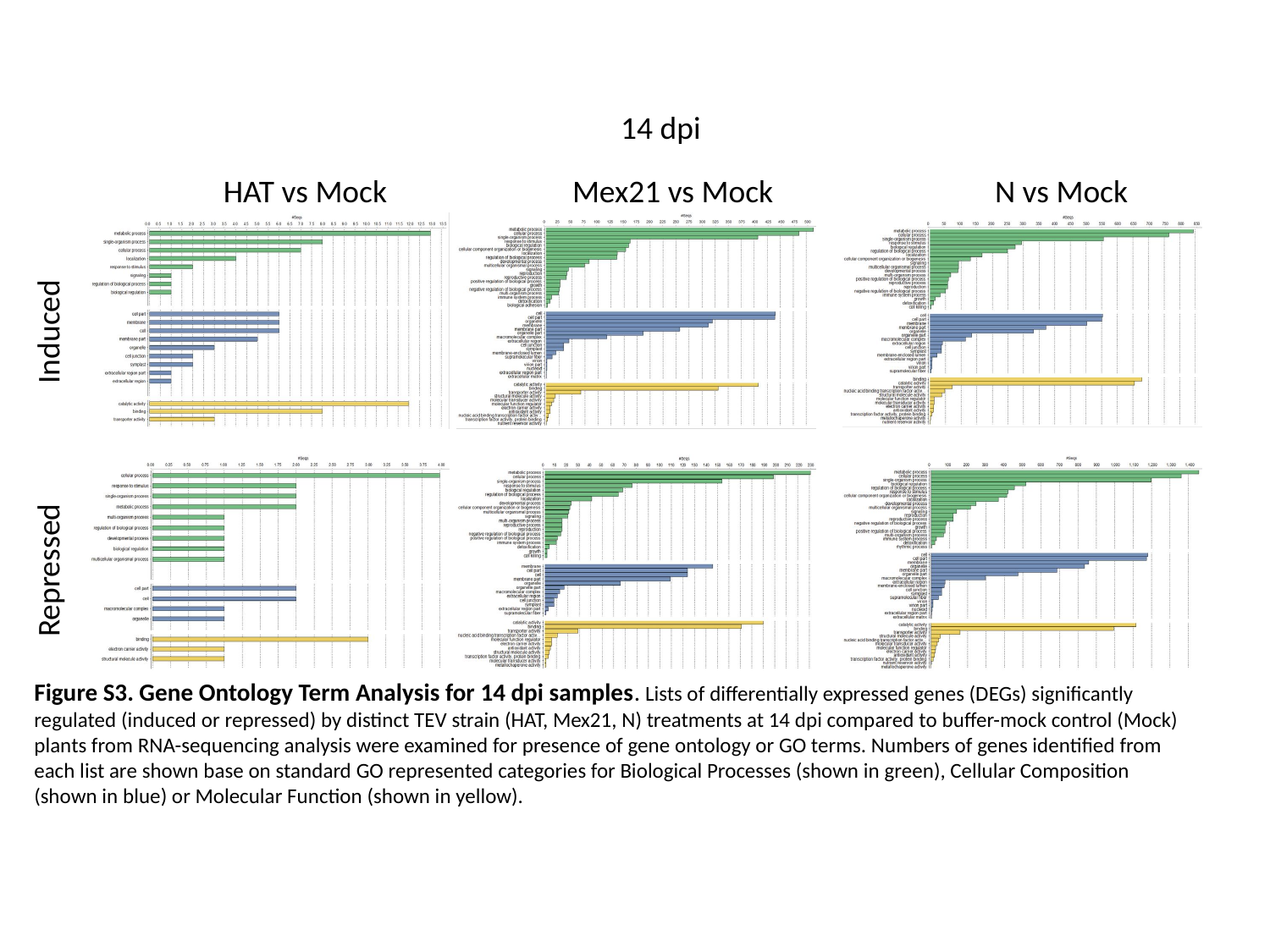

14 dpi
HAT vs Mock
Mex21 vs Mock
N vs Mock
Induced
Repressed
Figure S3. Gene Ontology Term Analysis for 14 dpi samples. Lists of differentially expressed genes (DEGs) significantly regulated (induced or repressed) by distinct TEV strain (HAT, Mex21, N) treatments at 14 dpi compared to buffer-mock control (Mock) plants from RNA-sequencing analysis were examined for presence of gene ontology or GO terms. Numbers of genes identified from each list are shown base on standard GO represented categories for Biological Processes (shown in green), Cellular Composition (shown in blue) or Molecular Function (shown in yellow).
